# Community Determinants of Physical Growth and Cognitive Development among Indian Children in Early Childhood: A Multivariate Multilevel Analysis

**DOI:** 10.3390/ijerph17010182

**Published:** 2019-12-26

**Authors:** Jongho Heo, Aditi Krishna, Jessica M. Perkins, Hwa-young Lee, Jong-koo Lee, S.V. Subramanian, Juhwan Oh

**Affiliations:** 1 National Assembly Futures Institute, 1 Uisadang-daero, Seoul 07233, Korea; joheo@nafi.re.kr; 2 JW Lee Center for Global Medicine, Seoul National University College of Medicine, Seoul National University College of Medicine, 71 Ihwajang-gil, Seoul 110-810, Korea; 3 Centre for Global Child Health, The Hospital for Sick Children, 525 University Avenue, Toronto, ON M5G 2L3, Canada; aditikrishna85@gmail.com; 4 Department of Human and Organizational Development, Peabody College of Education and Human Development, Vanderbilt University, 230 Appleton Place, Nashville, TN 37203, USA; jessica.m.perkins@vanderbilt.edu; 5 Takemi Fellow at the Department of Global Health and Population, Harvard T.H. Chan School of Public Health, 677 Huntington Ave, Boston, MA 02115, USA; diana0224@gmail.com; 6 Department of Family Medicine, Seoul National University College of Medicine, 71 Ihwajang-gil, Seoul 110-810, Korea; docmohw@snu.ac.kr; 7 Harvard Center for Population and Development Studies, Harvard T.H. Chan School of Public Health, 9 Bow Street, Cambridge, MA 02138, USA; 8 Department of Social and Behavioral Sciences, Harvard T.H. Chan School of Public Health, 677 Huntington Ave, Boston, MA 02115, USA

**Keywords:** community characteristics, local programs, health resources, Young Lives, India

## Abstract

Inadequate child physical growth and cognitive development share common individual-level risk factors. Less understood is how outcomes co-cluster at the community level and to what extent certain community-level characteristics influence the clustering. This study aims to quantify the extent to which child growth and development co-occur across communities, and to identify community-level characteristics associated with the clustering of the two development dimensions. We used longitudinal data from 1824 children (aged 5 years) across 98 communities in Andhra Pradesh, India in round 2 (2006) of the Young Lives study, who were followed up 3 years later in round 3 (2009). A multivariate, multilevel statistical model was estimated wherein the responses were nested within individuals, and communities. We used z-scores of height-for-age, weight-for-age, Peabody Picture Vocabulary Test, and a mathematics test in 2009 as outcome variables. At the community level, we included compositional variables representing community characteristics while controlling for child socio-demographic characteristics at the individual level. At the community level, children’s physical growth and cognitive development were strongly correlated (coefficient: 0.55–0.76) and, even after controlling for individual-level covariables, a more pronounced correlation was shown at the community level than individual level correlation. Greater local healthcare resources were associated with better physical growth. More local programs run by government and NGOs/charities were associated with higher child language skills. Local social problems were inversely associated with math scores. Our study showed that physical growth and cognitive development tended to be clustered and co-occurred within communities as well as individual children.

## 1. Introduction

Childhood is a critical period for proper development of the body and brain [1]. However, more than 200 million children in developing countries experience developmental deficits [2]. Inadequate child development is a critical problem, as these children will be likely to subsequently have poorer levels of educational achievement, poorer health in subsequent life stages, a lower probability of employment, and lower earnings [3,4,5]. In addition, impaired child development, in turn, negatively affects developmental outcomes among future generations through lasting effects on educational attainment and livelihoods. Thus, improving children’s developmental potential plays an important role in cutting the chain of intergenerational poverty transmission [3,6].

It is well known that inadequate physical growth and cognitive development share common risk factors such as poverty, malnutrition, infectious diseases, lower parental SES, and family-environmental adversity [7,8,9,10,11]. Although the community context has been highlighted as a critical level for interventions [12,13], much research on child growth and development has focused on individual-level factors especially within developing countries. Further, existing research assessing links between poor community conditions and adverse child development has dealt with either physical or cognitive outcomes, but not both [14,15,16,17,18,19]. Few studies have examined clustering of child physical growth and cognitive outcomes co-occurrence at the community level nor considered the shared environmental factors influencing both dimensions of child development.

In this paper, we bring together two bodies of literature on child physical growth and cognitive development using a multivariate multilevel analytical approach that enables us to jointly regress multiple outcomes on multilevel explanatory variables [20]. Multivariate analysis is the analysis that assesses more than two outcomes simultaneously, which is frequently confused with multiple or multivariable regression analyses in the literature [21]. Taking a multivariate multilevel approach uniquely allows us to examine community-level predictors as well as individual level predictors that simultaneously affect both physical growth and cognitive development, which could not be done if regression models were separate for each outcome. As a result, we can explore the extent to which communities simultaneously affect these two child development dimensions, adjusting for community-level compositional effects. Given that community dynamics are complex social processes, we pose an ecological and systemic perspective embracing environmental, psychological, and material characteristics of communities [22,23], rather than using the urban–rural divides or a single community dimension as community-level variables. If our study finds that inadequate child physical growth and cognitive development are clustered within communities, our findings would support investments in community-level interventions to prevent the co-occurrence of these two problems [21].

We chose data from India for our study due to the persistently high rates of stunting and underweight children in India [24]. A recent report showed that about 48% of children and more than 44% of children in India were stunted and underweight, respectively, during 2009–2013 [24]. The aim of this study is (1) to investigate the variation, covariation, and correlation of these two outcomes at the individual and community level, and (2) to identify community-level characteristics associated with clustering of child physical growth and cognitive development.

## 2. Materials and Methods

### 2.1. Study Population

The Young Lives study is a longitudinal cohort study designed to better understand the etiologies and consequences of childhood poverty and provide evidence for designing effective policies in developing countries [25]. The study contains information about environmental and social realities of households and communities through surveys for children, their caregivers, teachers, and community representatives and qualitative interviews with a subset of respondents to study child health and well-being. The study has followed about 12,000 children and their households over 15 years in four low- and middle-income countries: Ethiopia, Peru, Vietnam, and India (Andhra Pradesh, divided into two in June 2014: Andhra Pradesh and Telangana). In each country, round 1 of the survey was carried out in 20 sentinel sites in 2002 with two cohorts of children: a younger cohort (aged between 6 and 18 months; born in 1994–1995) and an older cohort (aged between 7.5 and 8.5 years; born in 2001–2002). Rounds 2 and 3 were conducted in 2006–2007 and in 2009–2010, respectively, with low attrition rates (0.50–3.52%) [26].

For this study, we used round 2 and 3 data collected in 2006–2007 and 2009–2010 from the younger cohort in India. One hundred households with children in the younger cohort were randomly selected for all the households in each sentinel site. One child per household was then randomly chosen as the index child, resulting in 2000 children surveyed in India. Detailed information about the Young Lives Study can be found elsewhere [25,27]. The study sample comprises children surveyed in round 2 who have a complete set of information on all individual- and community-level variables and outcome variables in round 3. Out of the original sample of 1950 children in Round 2, 1931 children were followed-up in round 3. Children with missing information on outcomes and co-variates at the individual level were dropped (n = 20, 1.0%). Outliers in outcomes based on the WHO standards (WHZ ± 5; HAZ ± 6) [28] were also dropped (n = 87, 4.5%). Thus, a total sample of 1824 children were used in this study. Ethical approval was not required for the study as we used secondary data that is publicly available.

### 2.2. Outcome Measures

Outcome variables were derived from round 3 (aged 8 years). Among the standardized anthropometric outcomes, we used height-for-age z-score (HAZ) and weight-for-age z-score (WAZ). Height-for-age represents the accumulated consequences of physical growth, which would not be expected to change in a short time period [29]. Low height-for-age is frequently found to be associated with poor overall economic conditions, especially mild to moderate, chronic or repeated infections, as well as inadequate nutrient intake [30]. Height and weight were collected by trained staff during the survey in respondents’ homes. They were converted into normalized HAZ and WAZ by using the WHO Anthro-Software package [31].

To measure the cognitive development of a child, the Peabody Picture Vocabulary Test (PPVT) version III and a mathematics test was used [32]. PPVT is a widely-used test of a child’s receptive language skills [32,33]. For 204 items, a child selects the picture that best represents the meaning of a stimulus word presented orally by the examiner. The test is conducted individually in an un-timed manner. For the Young Lives study in India, the test was translated into the main languages by the local team, verified by a local expert, and administered by fieldworkers [34]. A mathematics test was conducted using 29 items on counting, number discrimination, knowledge of numbers, and basic operations with numbers. To avoid a bias resulting from poor reading skills, the test was administered by interviewers who read questions aloud [34]. We used z-scores of PPVT scores (PPVTZ) and the mathematics test scores (MATHZ), and standardized raw scores within the country.

### 2.3. Individual-and Household-Level Characteristics

Individual-level variables include: age, ethnicity, caregiver’s education, mother’s height, family structure, birth order, mother’s age at birth, and wealth index. Ethnicity was measured as four categories: Backward castes (reference), Scheduled castes, Scheduled tribes, and other categories from Hindu, Muslim, Buddhist, and Christian. Caregiver’s education was categorized by no education (reference), primary schooling, and post-secondary or above. Family structure category included living with both parents (reference) and living with single or no parent. Birth order was operationalized as first (reference), second, or third and greater. Mothers’ age at birth was categorized into 20 years or below (reference); 21–30 years; and 31+ years. The wealth index was constructed from three indices: housing quality (main material of walls, roof, and floor, and household density); access to services (electricity, drinking water source, sanitation facility, fuel for cooking); and ownership of consumer durables (list of country-specific household items) [35]. Continuous variables, age (in months), mother’s height (in cm), and wealth index, were standardized within the country, and other independent variables were entered as dummy variables in our models.

### 2.4. Community-Level Characteristics

Communities in urban areas are defined as municipal wards identified by census codes, which are often at the level of policy allocation. In rural areas, villages and their associated hamlets were defined as communities [36]. A community is a meso-level area that is between the village level and the sentinel level, comprising 5.6 villages/hamlets on average [36]. Community contextual information was retrieved from two information sources: secondary data and key community leaders [37]. If secondary data of community-level information such as census records existed, the information was entered in the questionnaire before the field survey. The field supervisor administered the community survey through interviews with key community leaders contacted during the community entry process. Key informants included municipal/community leaders, government officials, health workers, teacher/school principals, leaders of women’s groups and religious leaders [37].

Community-level variables were operationalized using questions from community dataset (see Supplementary Table S1). We used five modules from the survey to identify community-level characteristics: (1) local pollution, (2) local social problems, (3) accessibility to local services, (4) local programs run by the government and NGOs/charities, and (5) local healthcare resources. Local pollution included 14 questions about pollution of water, land, air, and other types. Local social problems included nine questions about whether certain kinds of crime were problems in the locality. The accessibility to local services module asked whether recreational, religious, communicational, public, and private sector services were available at the time. The local programs run by the government and NGOs/charities module comprised questions on whether such programs were operating at the time. The module consisted of 26 questions covering food assistance programs, employment generation programs, education programs, health programs, infrastructure programs, and credit programs. The local healthcare resources module asked about the availability of various health facilities and health workers. All the questions in the modules were asked as dummy variables. A “yes” response to the questions was coded as one, and was otherwise coded as 0. Community-level variables were used as continuous variables by summing these scores by each module.

### 2.5. Statistical Models

**Figure 1:**
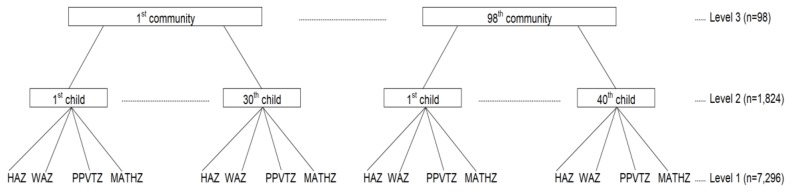
Multivariate multilevel structure of responses (HAZ, WAZ, PPVTZ, MATHZ) at level 1 nested within children at level 2 nested within communities at level 3. Note: HAZ: height-for-age z-score, WAZ: weight-for-age z-score, PPVTZ: Peabody Picture Vocabulary Test z-score, MATHZ: mathematics test z-score.

To estimate the variance of each outcome and the covariance of the two outcomes across individuals within communities, we fitted a multivariate, multilevel linear regression [20,38]. At level 1, multiple responses (HAZ, WAZ, PPVTZ, and MATHZ) from each individual were treated as repeated measures nested within that respondent. For the whole population, 7296 responses at level 1 were nested within 1824 children at level 2, who were in turn nested in 98 communities at level 3 (Figure 1). At the individual and community levels, we estimated variance and covariance, and correlation. Covariance shows how the two outcomes varied in the same direction. If covariance is greater (smaller) than 0, the outcomes varied in the same (opposite) direction. Based on the covariance, we assessed community-level correlation to examine whether communities with a high proportion of low HAZ and WAZ children also had a high proportion of low PPVTZ and MATHZ. Level-1 variation cannot be estimated because level 1 is needed solely to define the multivariate structure. We fitted three models by sequentially adding variables to the previous model to show the changes in variance, covariance, and correlations across the models. Initially we included no covariates and controlled individual- and community-level clustering effects (Model 1, null model); then added in individual-level variables (Model 2); and lastly community-level variables (Model 3, full model). We used multilevel software *MLwiN* (version 2.32) using the Stata (version 14) *runmlwin* command for model estimation.

### 2.6. Sensitivity Analysis

If children moved into a new community and were exposed to the community’s environment for a relatively short time, the environmental influences on their development might be less than for children who resided within one community for a long time. To address this concern, we conducted a sensitivity analysis comparing estimates of the original model to a model excluding children who moved into the community after the previous survey in round 1 (2002) (76 children, 4.1% of the study sample).

## 3. Results

Table 1 presents descriptive information of the family backgrounds of the sample children. More than 53% of caregivers had no formal education. The mean of mother’s height was 151.4 cm (SD 6.5). On average, communities had 2.7 pollution problems (SD 1.7), 1.8 social problems (SD 1.1), 11.5 accessible local services (SD 7.1), 27.9 programs run by governments and NGOs/charities (SD 7.1), and 2.1 kinds of health resources (SD 2.6).

Supplementary Table S2 shows results from the sensitivity analysis to examine whether there were differences in estimates between the original model and a model only for children that lived in the same community after the previous 2002 survey. Results from the sensitivity analysis suggest that there was little difference between the models.

We found individual- and community-level factors associated with the outcomes, in terms of magnitude and significance (Table 2). There were no common community factors that were associated with both physical growth and cognitive development. Local healthcare resources were associated with increasing physical growth. Local programs run by the government and NGOs/charities were only associated with better cognitive development. Local social problems were associated with lower math scores. Increasing child age was negatively associated with WAZ, but positively associated with PPVTZ and MATHZ. Higher caregivers’ education was more likely to have better physical growth and cognitive development than those whose caregivers had no education. Birth order of third or greater was inversely associated with better physical growth and cognitive development than the first child. Mother’s older age and greater height were positively associated with better physical growth. Wealth index was positively associated with both physical growth and cognitive development.

Table 3, Table 4 and Table 5 provide variance, covariance, and correlations between HAZ, WAZ, PPVTZ, and MATHZ at the individual and community level to demonstrate how both outcomes varied, covaried, and correlated across the levels. In Table 3, when individual-level covariables were included (Model 2), the community-level variations in HAZ and WAZ decreased by 78.5% (0.14 vs. 0.03) and 92.8% (0.14 vs. 0.01), respectively. PPVTZ and MATHZ decreased by 35.3% (0.17 vs. 0.11) and 36.4% (0.22 vs. 0.14), respectively. Further, when community characteristics were included in Model 3, variations in outcomes remained significant. In Table 4, the community-level covariance between physical growth (HAZ and WAZ) and cognitive development (PPVT and MATHZ) decreased by including individual-level covariables, resulting in a covariance range of 0.012–0.098 (between physical growth and language skills) and 0.001–0.129 (between physical growth and mathematics scores). In Table 5, correlations between physical growth and cognitive development after controlling for individual-level covariables varied according to combinations between outcome indicators (correlation coefficients: 0.18–0.71 (at community level model 2), 0.11–0.17 (at individual level model 2)). Notably, a more pronounced correlation between physical growth and cognitive development was shown at the community level (vs. individual level correlation).

## 4. Discussion

We used a multivariate multilevel approach to (1) investigate the variation, covariation, and correlation in children’s physical growth and cognitive development using the Young Lives study India dataset, and (2) identify community-level characteristics associated with the two outcomes. We found a stronger correlation between physical growth and cognitive development at the community level than at the individual level. This may suggest that children’s physical growth and cognitive development tend to cluster within communities even after accounting for natural lottery cluster within a child (i.e.,: gene inheritance influence). Further, physically delayed children were also more likely to be cognitively delayed. In addition, we found significant associations between several community-level characteristics and outcomes. Local pollution was associated with worse cognitive development. Children living in communities with more local healthcare resources were likely to have better physical growth. Local programs run by the government and NGOs/charities were associated with better receptive language skills in children.

A novel aspect of our study was to quantify the co-occurrence of the physical growth and cognitive development of the child at the individual level and the community level simultaneously as well as separately. We found correlations in patterning of between-community variations of the outcomes. A more pronounced correlation was observed at the community level, suggesting that a larger extent of the variation in co-occurrence was between communities. This finding implies that communities have the potential to simultaneously promote physical growth and cognitive development in children. This is the first study to quantitatively demonstrate the simultaneous importance of the community-level for connected outcomes.

Second, the number of local healthcare resources was associated with physical growth, a result supported by previous studies suggesting that regional healthcare resources may impact child nutritional status and child height by providing access to treatment for common infectious diseases especially in poor settings [39,40]. Children may benefit from community-level healthcare resources enough to gain more weight. However, our findings of community-level variation in child development outcomes may reflect inequity in India due to an imbalance in resource allocation, inadequate physical access to healthcare facilities and human resources, and access to antenatal care and infant and young child immunizations [41,42]. Further, many rural practitioners are not formally trained or licensed [43].

Third, we found that children’s cognitive development was positively associated with local programs run by the government and NGOs/charities. Existing studies showed that local social protection and universal education programs supporting impoverished families were an effective approach to reducing poverty in low-income countries, consequently also supporting child development [44]. A few studies have provided evidence of the importance of community-level programs and child development using the Young Lives data. For example, a Peru study reported that an early child development program promoted child physical growth and cognitive development [45]. In addition, the Ethiopian social assistance program known as the Productive Safety Net Program decreased child work for pay, reduced child labor time, and consequently increased the highest children’s grade [46]. Finally, an Indian study reported that the Midday Meal Scheme implemented as a security net for children boosted cognitive scores as well as buffered adversity from malnutrition [47].

Fourth, our findings of associations between local social problems and worse child cognitive development are consistent with previous studies showing that danger and crime in the community may adversely influence child development [48,49]. Previous studies explained that children living in communities that lack informal control or collective efficacy may have difficulty accessing resources such as after school programs or extracurricular activities that might foster child cognitive developments [50,51]. Parents may be also less likely to allow their child to play outside in such communities [52,53], and consequently, reduce opportunities to enhance their children’s cognitive skills.

Our study has several limitations. First, we adopted a longitudinal approach to ensure qualified inferences regarding the cause-effect relations; however, causality cannot be inferred due to unmeasured confounding. Second, we cannot eliminate the possibility of other unmeasured mediators, even though we included several covariates in order to reduce confounding. Third, we cannot avoid the bias caused by non-random attrition. Similar to other longitudinal surveys, disadvantaged households were more likely to drop out in the Young Lives Survey even though attrition rates were relatively small when compared with other longitudinal studies in developing countries [26]. Fourth, our result has the limitation of generalizability, since the survey only included children living in Andhra Pradesh and Telangan. Last, we were not able to measure the quality dimension as well as the quantity aspect of community characteristics due to the limitation of secondary survey data. For example, the survey asked only whether a public hospital was currently available in the locality, rather than the number or quality of available hospitals in the locality. Future longitudinal studies should further investigate whether community characteristics also have long-term effects on child physical growth and cognitive development.

## 5. Conclusions

In conclusion, we found that child physical growth and cognitive development were clustered within communities. The correlation between physical growth and cognitive development at the community level largely remained even after adjusting for individual-level covariates. We also identified community characteristics that were significantly associated with these outcomes. Our study strongly supports and legitimizes a community-unit intervention approach (in addition to targeting individuals) to tackle inadequate child physical growth and cognitive development in resource limited societies such as India.

## Figures and Tables

**Table 1:** Information for 5-year-old children’s individual/family backgrounds and community characteristics in Andhra Pradesh, India from Young Lives 2006.

Level 1: Multivariate Outcomes		Mean	SD
Height-for-age (z-score)		−1.5	1.0
Weight-for-age (z-score)		−1.9	1.0
Peabody Picture Vocabulary Test (z-score)		−0.01	1.0
Mathematics test (z-score)		−0.001	1.0
Level 2: Individual Characteristics (n = 1625)		N	%
Sex	Boys	968	53.1
	Girls	856	46.9
Ethnicity	Scheduled castes	325	17.8
	Scheduled tribes	233	12.8
	Backward castes	882	48.4
	Other categories	384	21.1
Caregiver’s education	No education	984	53.9
	Primary	782	42.9
	Post-secondary or above	58	3.2
Family structure	Living with both parents	1789	98.1
	Living with single or no parent	35	1.9
Birth order	First	703	38.5
	Second	712	39.0
	Third or later	409	22.4
Mother’s age at birth	20 or below	551	30.2
	21–30	1175	64.4
	31 or above	98	5.4
		Mean	SD
Age in months		95.8	3.8
Mother’s height		151.4	6.1
Wealth index		0.5	0.2
Level 3: Community characteristics (n = 94)		Mean	SD
Local pollution problems		2.7	1.7
Local social problems		1.8	1.1
Access to local services		11·5	4.1
Local programs run by government and NGO/charity		27.9	7.1
Local healthcare resources		2.1	2.6

**Table 2:** Estimates (coeff.) and standard errors (SEs) from the fixed part of multivariate 3-level linear models for individual- and community-level variables.

	HAZ		WAZ		PPVTZ		MATHZ	
	Coeff.	SE	Coeff.	SE	Coeff.	SE	Coeff.	SE
Community-level variables								
Local pollution problems	−0.001	0.02	0.02	0.02	0.01	0.03	−0.04	0.03
Local social problems	−0.003	0.03	−0.001	0.02	0.002	0.04	−0.08 *	0.04
Access to local services	−0.004	0.01	0.002	0.01	0.01	0.01	0.004	0.01
Local programs run by government and NGO/charity	−0.001	0.005	0.0004	0.004	0.01 *	0.01	0.02 **	0.01
Local healthcare resources	0.04 **	0.01	0.03 *	0.01	−0.02	0.02	−0.01	0.02
Individual-level variables								
Age (in months)	−0.02	0.02	−0.08 ***	0.02	0.07 ***	0.02	0.11 ***	0.02
Sex (reference: girls)								
Boys	−0.05	0.04	−0.16 ***	0.04	0.27 ***	0.04	0.08 *	0.04
Ethnicity (reference: Backward castes)								
Scheduled castes	0.00	0.06	0.07	0.06	0.08	0.06	−0.02	0.06
Scheduled tribes	−0.13	0.08	0.08	0.08	−0.10	0.09	−0.28 ***	0.08
Other categories	0.11	0.06	0.22 ***	0.06	0.02	0.06	0.01	0.06
Caregiver’s education (reference: no education)								
Primary or below	0.13 *	0.05	0.08	0.05	0.17 ***	0.05	0.33 ***	0.05
Post-secondary or above	0.33 *	0.14	0.44 **	0.14	0.52 ***	0.13	0.66 ***	0.13
Family structure (reference: living with both parents)								
Living with single or no parent	−0.07	0.15	−0.03	0.16	0.05	0.15	0.29 *	0.14
Birth order (reference: first)								
Second	−0.12 *	0.05	−0.11 *	0.05	−0.09	0.05	−0.04	0.05
Third or greater	−0.36 ***	0.06	−0.37 ***	0.07	−0.22 ***	0.06	−0.22 ***	0.06
Mother’s age at birth (reference: 20 or below)								
21–30	0.20 ***	0.05	0.17 ***	0.05	0.19 ***	0.05	0.08	0.05
31 or above	0.37 ***	0.11	0.23 *	0.11	0.14	0.10	0.14	0.10
Mother’s height	0.24 ***	0.02	0.16 ***	0.02	−0.01	0.02	0.03	0.02
Wealth index	0.18 ***	0.03	0.21 ***	0.03	0.18 ***	0.03	0.21 ***	0.03

Note: HAZ: height-for-age z-score, WAZ: weight-for-age z-score, PPVTZ: Peabody Picture Vocabulary Test z-score, MATHZ: mathematics test z-score, * *p* < 0.05, ** *p* < 0.01, *** *p* < 0.001.

**Table 3:** Individual- and community-level variance in height-for-age z-score (HAZ), weight-for-age z-score (WAZ), Peabody Picture Vocabulary Test z-score (PPVTZ), and math test z-score (MATHZ).

Random Effects	Model 1		Model 2		Model 3	
	Estimate	SE	Estimate	SE	Estimate	SE
Level 3 (Community)						
HAZ	0.14	0.03	0.03	0.01	0.03	0.01
WAZ	0.14	0.03	0.01	0.01	0.01	0.01
PPVTZ	0.17	0.03	0.11	0.02	0.10	0.02
MATHZ	0.22	0.04	0.14	0.03	0.11	0.02
Level 2 (Individual)						
HAZ	0.90	0.03	0.80	0.03	0.80	0.03
WAZ	0.97	0.03	0.89	0.03	0.88	0.03
PPVTZ	0.82	0.03	0.74	0.03	0.74	0.03
MATHZ	0.76	0.03	0.66	0.02	0.66	0.02

Note: Model 1 is the null model; Model 2 includes individual-level variables (age in months, sex, ethnicity, caregiver’s education, family structure, birth order, mother’s age at birth and wealth index); Model 3 includes community-level variables (local pollution problems, local social problems, access to local services, local programs run by government and NGOs/charities, and local healthcare resources).

**Table 4:** Individual- and community-level covariance and standard errors (in parentheses) in height-for-age z-score (HAZ), weight-for-age z-score (WAZ), Peabody Picture Vocabulary Test z-score (PPVTZ), and mathematics test z-score (MATHZ).

	Model 1		Model 2		Model 3	
	PPVTZ	MATHZ	PPVTZ	MATHZ	PPVTZ	MATHZ
Level 3 (Community)						
HAZ	0.095 (0.024)	0.098 (0.026)	0.012 (0.011)	0.001 (0.012)	0.017 (0.011)	0.003 (0.010)
WAZ	0.117 (0.025)	0.100 (0.026)	0.028 (0.011)	0.008 (0.011)	0.029 (0.010)	0.010 (0.017)
Level 2 (Individual)						
HAZ	0.126 (0.021)	0.188 (0.020)	0.088 (0.019)	0.129 (0.018)	0.086 (0.019)	0.129 (0.018)
WAZ	0.126 (0.022)	0.179 (0.021)	0.098 (0.020)	0.129 (0.019)	0.097 (0.020)	0.129 (0.019)

Note: Model 1 is the null model; Model 2 includes individual-level variables (age in months, sex, ethnicity, caregiver’s education, family structure, birth order, mother’s age at birth and wealth index); Model 3 includes community-level variables (local pollution problems, local social problems, access to local services, local programs run by government and NGOs/charities, and local healthcare resources).

**Table 5:** Individual- and community-level correlation in height-for-age z-score (HAZ), weight-for-age z-score (WAZ), Peabody Picture Vocabulary Test z-score (PPVTZ), and mathematics test z-score (MATHZ).

	Model 1		Model 2		Model 3	
	PPVTZ	MATHZ	PPVTZ	MATHZ	PPVTZ	MATHZ
Level 3 (Community)						
HAZ	0.61	0.55	0.20	0.19	0.11	0.10
WAZ	0.76	0.57	0.21	0.18	0.12	0.17
Level 2 (Individual)						
HAZ	0.15	0.23	0.11	0.17	0.11	0.17
WAZ	0.14	0.21	0.12	0.17	0.12	0.17

Note: Model 1 is the null model; Model 2 includes individual-level variables (age in months, sex, ethnicity, caregiver’s education, family structure, birth order, mother’s age at birth and wealth index); Model 3 includes community-level variables (local pollution problems, local social problems, access to local services, local programs run by government and NGOs/charities, and local healthcare resources).

## Supplementary Materials

The following are available online at https://www.mdpi.com/1660-4601/17/1/182/s1, Table S1: Community-level characteristics from Indian data, Young Lives round 2, 2006, Table S2: The result of sensitivity analysis.

## Abbreviations

HAZ	height-for-age z-score
MATHZ	mathematics test scores
PPVT	Peabody Picture Vocabulary Test
PPVTZ	z-scores of the Peabody Picture Vocabulary Test scores
WAZ	weight-for-age z-score
