# Supplementary material for: Community Determinants of Physical Growth and Cognitive Development among Indian Children in Early Childhood: A Multivariate Multilevel Analysis
Source: Int J Environ Res Public Health. 2019 Dec 26;17(1):182. doi: 10.3390/ijerph17010182 (PMC6981961; doi:10.3390/ijerph17010182)
Supplement: Supplementary file 1 [file ijerph-17-00182-s001.pdf]

Table S1. Community-level characteristics from Indian data, Young Lives round 2, 2006

1. Local Pollution (n=14)

Is this a problem in locality?
**Water pollution**
Industrial waste deposited or dumped at (or in) water sources
Mining waste deposited or dumped at (or near) water sources
Local families garbage dumped at (or in) these water sources
Animal feces at (or in) water sources
**Land pollution**
Wasteland (non-regulated space) where firms dump garbage
Wasteland where local families dump garbage
Wasteland where families from other localities dump garbage
Animals' excrements on the streets (of the locality)
**Air pollution**
Garbage burning
Industrial activity
Trucks, cars, and the like passing by locality
Use of pesticides, fertilizers in local agricultural lands
**Other types of pollution**
Noise pollution (motor vehicles, airports, factories)
Standing water, open drains (with the consequent proliferation of mosquitoes, rats and other animals that threaten local population's health)

2. Local Social Problems (n=9)

Is this a problem in locality?
Theft / robbery
Cattle/livestock theft
Prostitution in the local area
Juvenile gangs
Local adult bands/groups rivalry (political, ethnic, economic-wise)
Illegal drugs selling in the local area
Drug-addicts in the local area (either residents or outsiders consuming illegal drugs in the local area)
Alcoholism
Violent crimes

3. Access to Local Services (n=26)

Is the following service currently available at locality?
Recreational areas: children's playgrounds<br>-space exclusively assigned for little children to play<br>(it might be an open field as long as it is clean and safe/secure)
Recreational areas: football (soccer) field, volleyball field, etc.<br>-space exclusively assigned for children and adolescents to exercise/practice a sport<br>(it might be an open field as long as it is clean and safe/secure)
Recreational areas: camping zones of family recreational areas
Movie theatre / public place for playing films
Video games

Cock fighting arenas / bull fighting arenas or other animal fighting arenas
Space for fairgrounds / circus (temporary recreational activities)
Slot machines / casino
Religious institutions, churches
Public telephones, community telephone, rural telephone.
Private telephone (at least one connection)
Public internet cabin
Electricity (at least one connection)
Drinking water (at least one connection)
Sewerage (at least one connection)
Police station
Non-professional judge
Professional judge
Nationalized banks
Other private banks
Local government credit/savings schemes
Primary agricultural cooperative societies
Anganwadi Center for Integrated Child Development Services (ICDS)
Regulated market
Fair price/ Public distribution system (PDS) shop
Veterinary hospital

4. Local Programs Run by Government and NGO/Charity (n=60)

Does this program currently operate at locality?

Food assistance programs/employment generation programs

National Mid-day meal scheme: school meal program run by the government of India
Public distribution system (PDS): Indian food security system
Antyodaya Anna Yojana (AAY): governmental foods subsidy scheme for the poorest
Annapurna Scheme: food security program for senior citizens

Education programs

Rural education scholarship
Free Text-Book
School feeding program (ICDS)
Free Transport and Free Tuition Fee programme
Education Guarantee Scheme under Sarva Shiksha Abhiyan (SSA)/Alternative School
Residential Schools run by Social Welfare departments

Health programs

Health insurance
Central Rural Sanitation Program (CRSP) (ISL)
Swajala dhara (Drinking Water)

Infrastructure programs

Soil & Water Conservation Program

Other programs

Food for work
Agricultural extension services
Widow pensions

National Family Benefit Scheme (NFBS)
National Maternity Benefit Scheme (NMBS)
Girl Child Protection Scheme
Indira Awas Yozana (IAY)
Credit Cum Subsidy Scheme for Rural Housing (CCSSRH)
National Old Age Pension Scheme (NOAP)
Sampoorna Grameena Rozgar Yozana (SGRY))
Swarnajayanti Gram Swarojgar yojana (SGSY)
Prime Minister's Rojgar Yojana (PMRY)
Pradhana Manthri Grameena Sadak Yojana
Developmentof WomenandChild in Rural Areas (DWACRA/DPIP/RPRP) Groups
Joint Forest Management (JFM)
Farmers Clubs
Watershed Development Works
Watershed (DPAP)
Watershed (Others)
Desert Development Program
Waste Land Development Program
National Rural Employment Guarantee Scheme
National Project on Bio-gas development
Integrated Rural Energy Program
Integrated Cattle Development Program
Integrated Dairy Development Program
Fish Farmer Development Program
Crop Insurance
Agriculture input subsidy/crop production program
Deepam Padhakam
Land Distribution (CLDP / Indira Prabha)
Adarana
Subsidy on purchase of Agrl. Machinery
Support of bonded and Child Labor
Free veterinary Camps
Cheyutha (for Disabled)
CM EY / Rajiv Yuva Shakti
Swadhar Programme (Child Trafficking)
Programmes of SC Corporation
Programmes of ST Corporation
Programmes of BC Corporation
Programmes of Minority Corporation
Kasturba Gandhi Balika Vidhhyalalaya
National Child Labour Programme
Andhra Pradesh Special Police
APRLP

5. Local Healthcare Resource (n=21)

Is there currently ____________ available in locality?
**Health facility**
Public hospital
Private hospital

State run health center (PHC)
Private clinic
Public clinic (sub center)
Health center (private)
Pharmacy
Family planning clinic (public)
Family planning clinic (private)
Private maternity home
Health post
**Health workers**
Trained midwife
Traditional birth attendant
Traditional healer
Curioso
Village health worker
Auxiliars/Technician nurses/Medical care assistant
Trained nurse
Social worker
Mental health worker
General physician
Specialist physician (pediatrician, gynecologists, psychiatrist, etc.)
Anesthetist
Dentist
Blood Banker

Table S2. The result of sensitivity analysis

	HAZ		WAZ		PPVTZ		MATHZ	
	Coefficient	SE	Coefficie	SE	Coefficie	SE	Coefficie	SE
Local pollution problems	-0.001	0.02	0.02	0.02	0.01	0.03	-0.04	0.03
Local social problems	-0.003	0.03	-0.001	0.02	0.002	0.04	-0.08*	0.04
Access to local services	-0.004	0.01	0.002	0.01	0.01	0.01	0.004	0.01
Local programs run by government and NGO/charity	-0.001	0.004	0.0004	0.004	0.01*	0.01	0.02**	0.01
Local healthcare resources	0.04**	0.01	0.03*	0.01	-0.02	0.02	-0.01	0.02
Age (in months)	-0.02	0.02	-0.08***	0.02	0.07***	0.02	0.11***	0.02
Sex (reference: girls)								
Boys	-0.05	0.04	-0.16***	0.04	0.27***	0.04	0.08*	0.04
Ethnicity (reference: Backward castes)								
Scheduled castes	0.004	0.06	0.07	0.06	0.08	0.06	-0.02	0.06
Scheduled tribes	-0.13	0.08	0.08	0.08	-0.10	0.09	-0.28***	0.08
Other categories	0.11	0.06	0.22***	0.06	0.02	0.06	0.01	0.06
Caregiver's education (reference: no education)								
Primary or below	0.13*	0.05	0.08	0.05	0.17***	0.05	0.33***	0.05
Post-secondary or above	0.33*	0.14	0.44**	0.14	0.52***	0.13	0.66***	0.13
Family structure (reference: living with both parents)								
Living with single or no parent	-0.07	0.15	-0.03	0.16	0.05	0.15	0.29*	0.14
Birth order (reference: first)								
Second	-0.12*	0.05	-0.11*	0.05	-0.09	0.05	-0.04	0.05
Third or greater	-0.36***	0.06	-0.37***	0.07	-0.22***	0.06	-0.22***	0.06
Mother's age at birth (reference: 20 or below)								
21-30	0.20***	0.05	0.17***	0.05	0.19***	0.05	0.08	0.05
31 or above	0.37***	0.11	0.23*	0.11	0.14	0.10	0.14	0.10
Mother's height	0.24***	0.02	0.16***	0.02	-0.01	0.02	0.03	0.02
Wealth index	0.18***	0.03	0.21***	0.03	0.18***	0.03	0.21***	0.03

Note: WAZ: weight-for-age z-score, PPVTZ: Peabody Picture Vocabulary Test z-score, *p<0.05, **p<0.01, ***p<0.001.
